# The neutrophil-to-lymphocyte ratio: a narrative review

**DOI:** 10.3332/ecancer.2016.702

**Published:** 2016-12-12

**Authors:** Sara Socorro Faria, Paulo César Fernandes, Marcelo José Barbosa Silva, Vladmir C Lima, Wagner Fontes, Ruffo Freitas-Junior, Agda Karina Eterovic, Patrice Forget

**Affiliations:** 1 Mastology Program, Federal University of Uberlandia (UFU), Avenida Engenheiro Diniz 1178, Reitoria 3º Andar Uberlândia, Minas Gerais, Brazil; 2 Department of Medical Oncology, AC Camargo Cancer Center, R Professor Antônio Prudente, 211, Liberdade, São Paulo 01509, Brazil; 3 Laboratory of Biochemistry of Protein Chemistry (Proteomics Research), University of Brasilia (UnB), Campus Universitário Darcy Ribeiro S/N (Asa Norte) 70910-900, Brazil; 4 Mastology Program, Federal University of Goias (UFG), Rodovia Goiania Nerópolis Km 12, Prédio da Reitoria, 74001-970, Goiania/GO, Brazil; 5 Department of Systems Biology, The University of Texas MD Anderson Cancer Center, 1515 Holcombe Boulevard, Houston, Texas 77030, USA; 6 Department of Anesthesiology and Perioperative Medicine, Universitair Ziekenhuis Brussel, laarbeeklaan 101, 1090 Brussels, Belgium

**Keywords:** breast cancer, inflammation, neutrophil-to-lymphocyte ratio, prognosis

## Abstract

Cellular-mediated inflammatory response, lymphocytes, neutrophils, and monocytes are increasingly being recognised as having an important role in tumorigenesis and carcinogenesis. In this context, studies have suggested that the neutrophil-to-lymphocyte ratio (NLR) can be used as an independent prognostic factor in a variety of cancers. Particularly in breast cancer, several studies have shown that a high NLR is associated with shorter survival. Because the NLR can be easily determined from the full blood count, it could potentially provide a simple and inexpensive test cancer prognosis. This review addresses the possibilities and limitations of using the NLR as a clinical tool for risk stratification helpful for individual treatment of breast cancer patients. The potential underlying phenomena and some perspectives are discussed.

## Introduction

Breast cancer (BC) is the most common form of cancer among women, representing approximately 23% of all tumours, and is a major global health concern [1]. Patient prognosis depends on multiple variables including patient-specific characteristics (e.g. performance status, age, and race), tumour biology (e.g. tumour size, nodal status, histologic grade), and response to systemic therapy (e.g. polymerase chain reaction, pCR, vs. no pCR) [2]. Inflammation is also likely to be an important marker of outcome in cancer. In cancer, clinical outcome may be influenced not only by the histopathological characteristics of the tumour itself, but also by the host response, including the inflammatory response. Therefore, predictive biomarkers reflecting the response of this disease to these agents may help to guide management.

Recent studies have confirmed the role of the host’s inflammatory responses in tumour development and progression of cancers including of breast cancer [3]. These works have shown that the secretion of cytokines and chemokines produced by both the tumour and associated cells (such as leukocytes) can contribute to the development of metastases [4]. A neutrophilic response is associated with poor prognosis, as it can inhibit the immune system by suppressing the cytotoxic activity of T cells, for example [5]. The presence of tumour-infiltrating lymphocytes (TILs) has been associated with a better response to cytotoxic treatment and prognosis in BC patients [6]. Similarly, haematological indices, such as neutrophil–to-lymphocyte ratio (NLR) [5], platelet-to-lymphocyte ratio (PLR) [7], lymphocyte-to-monocyte ratio (LMR) [8], and C-reactive protein (CRP) [9], have been to assess inflammatory response.

In this context, the neutrophilic inflammatory response has the potential to be an important marker of poor prognosis. NLR is an easily measured, reproducible, and inexpensive marker of subclinical inflammation. Additionally, NRL is indicative of an impaired cell-mediated immunity associated with systemic inflammation [4]. The prognostic role of NLR has been documented in multiple cancers, disease settings and treatments including malignancies of the colon [10], ovaries [11], urothelium [12], pancreas [13], and kidneys [14].

Although some studies have documented an association of elevated NLR in patients to a poor prognosis breast cancer [15–17], the overall evidence on the prognostic role of the NLR is relatively scarce. The purpose of this review was to examine the link between the NLR and the prognosis in breast cancer and to investigate the connection between NLR and clinical pathological factors.

## Prognostic factors for breast cancer

There is an ongoing search for new classification schemes, markers and additional prognostic factors to best determine the clinical outcome of BC stratify risk groups, identify accurately patients at risk or not of recurrence and those who respond or not the specific treatment. The main question to be answered is how to select and identify the best treatment in each case. One potential strategy involves the use of biomarkers. Currently, molecular, prognostic, and predictive factors that have been incorporated into the mammary pathological evaluation routine include: TNM staging, tumour size, the presence of lymph node alterations, histological type and grade, expression of hormone receptors such as oestrogen receptor (ER) and progesterone receptor (PR), amplification of the epidermal growth factor 2 gene (HER-2/Neu), overexpression of Bcl-2, mutations in *p53*, *BRCA1* and *BRCA2* genes, expression of cathepsin D, cyclin D1, and protein levels of Ki67 antigen (a cell proliferation marker). The presence and extent of carcinoma in situ, and age may also be used as the diagnosis and prognosis of the patients [18].

Non-small cell lung cancer (NSCLC), colorectal cancer, and breast cancer have been classically described as ‘non-immunogenic’. However, in recent classifications of breast cancer, using the multiple differential gene expression, there is evidence that certain types of breast cancer, such as triple negative (considered the most immunogenic BC subtype), show a high level of expression of activating genes in pathways involved in the immune response; the presence of infiltrating immune cells and lymphocytes (TILs) within the tumour microenvironment and genetic instability leading to increased number of mutations [19].

An exponential number of studies have found that an elevated neutrophil-to-lymphocyte ratio (NLR), reflecting, at least partially, the inflammatory response, was found to be an independent prognostic factor for adverse outcomes in several solid tumours, including BC [15]. Fortunately, determining the NLR requires a minimally invasive test (blood test) that allows the evaluation of tumour activity and its interaction with the microcirculation with good sensitivity and specificity (Table 1). Moreover, the parameters used to calculate NLR are based on data available in routine services; therefore, no additional costs are involved.

## Neutrophils and lymphocytes in cancer

The neutrophils may act as tumour-promoting leukocytes, capable of stimulating and suppressing tumorigenesis antitumour immune response; participate in metastatic cascade; are effectors of angiogenesis; promote leakage of tumour cells and endothelial cells into the circulation, therefore contributing to reroute the inflammatory response into a tumour-promoting direction [20]. Some immunocytes, as neutrophils, can secrete circulating vascular endothelial growth factor (VEGF) that increase the tumour development [21]; therefore, an elevated neutrophil count can stimulate tumour angiogenesis and contribute to disease progression, thus leading to a negative correlation between neutrophil density and patient survival.

Approaches such as the one described by Templeton *et al*. [5] have linked elevated neutrophil counts in blood with increased risk for metastasis in all disease subgroups and tumour sites. In patients with BC, increased neutrophil abundance predicts worse metastasis-specific survival [22]. The authors did not find significant differences in the frequencies and activation of various immune components as a consequence of neutrophil depletion, in cytotoxic T and natural killer’s cells [23]. In another model, Coffelt *et al*., [24] evaluated the role of neutrophils in different phases of the metastatic cascade, demonstrating that interleukin (IL)-1b elicits IL-17 expression from gamma delta (cd) T cells. In both studies, the authors hypothesise that neutrophils can promote metastasis via immune suppression.

The structural and cellular composition of the breast provides a unique microenvironment favourable for both tumour growth and localised inflammation. The host’s anticancer immune response greatly depends on lymphocytes that are distributed in specific areas. The tumour microenvironment in breast cancer is made by a variety of cells, including B and T lymphocytes non-neoplastic, plasma cells, oeosinophils, macrophages, mast cells, and fibroblasts. The T lymphocytes infiltrate in BC indicates immune imbalance characterised by a predominance of CD4^+^ T lymphocytes phenotypes T helper 2 (Th2) and regulatory T (Treg FoxP3^+^, CD4^+^, CD25^+^) [25]. In contrast, the presence of lymphocytes in the tumour is associated with better responses to chemotherapy and better prognosis in BC patients [6].

The clinical relevance of the interaction between neutrophils and lymphocytes inflammatory responses play critical role in carcinogenesis. Thus, the NLR can reflect the balance between the activation of the inflammatory pathway and the antitumour immune function in BC. Furthermore, increase in neutrophils count could be a consequence of cancer-associated inflammatory components, such as IL-6, tumour necrosis factor alpha (TNF-α) and granulocyte colony-stimulating factor [26]. On the other side, immune response associated with BC concerns primarily lymphocytes, with a depressed cellular immunity, a decreased number of CD4 lymphocytes and an increased CD8 lymphocytes activation [26]. Inflammation can generate not only a cancer-prone microenvironment but also systemic changes in the host that accelerate cancer growth. An elevated NLR can cause neutrophilia linked to tumour granulocyte colony-stimulating factor (GCSF), can accelerate tumour development and increase in plasma cytokines IL-6 and TNF-α), while the lymphopenia is associated with diseases’ severity and immune escape of tumour cells from tumour-infiltrating lymphocytes [27].

## NLR in breast cancer—impact on overall survival and disease-free survival

An increase in NLR has been reported to correlate with poor prognosis in patients with malignant tumours [5]. In BC, the NLR have been associated with survival in breast cancer [16, 17]. Previous studies suggest that the levels of systemic inflammation are indicative of decreased survival, mainly because immunocytes within the tumour microenvironment play a role in tumour development and in the survival of neoplastic cells [5].

The mechanism that underlies the association between the high cut-off NLR and poor prognosis has not been elucidated. Systemic inflammatory phenomena would be reflected in increases in the neutrophil counts, and these could induce tumoural agressiveness and cancer progression [5]. Inflammatory components can inhibit the immune response by suppressing the cytotoxicity of the immunocytes, can promote tumour neo-angiogenesis, invasion of adjacent tissues, and metastatic progression by recruiting regulatory T lymphocytes and cytokine secretion [27].

Meta-analyses have shown an association between a high NLR and worse long-term outcomes, after treatment, in various types of cancers [28–30]. There is no consensus cut-off, or threshold, value for the NLR to reliably stratify the risk of recurrence or the risk of mortality. It has been shown by Azab *et al*. [30] in a cohort of 437 women with BC that a high pretreatment NLR is a significant risk factor for an increased mortality, regardless of the chemotherapy regiment. In this study, patients with NLR > 3.3 were older and had advanced disease. In addition, NLR cut-off > 3.3 remained an independent significant predictor.

Dirican *et al*. [16] focused on the performance of the preoperative NLR as a prognostic factor in 1527 patients with BC with a follow-up of nearly 6 years. They used a cut-off value of 4, and the NLR was again independently associated with disease-free survival (DSF) and overall survival (OS). Krenn-Pilko *et al*. [31] reported in 762 European BC patients that NLR > 3 is an independent risk factor for poor disease-free survival but not of overall survival.

It is important to mention that ethnicity, cultural challenges, different genetic structure, several inflammatory status, and other pathological factors described in the studies above can partially explain the variation in survival among patients. Additionally, the median value of NLR used as a cut-off to stratify high and low NLR groups, which varies across studies and the sample size may be associated with a kind of selection bias. In this context, a meta-analysis of five studies has demonstrated that elevated pretreatment NLR, with the cut-off values ranged between 2.0 and 4.0, was associated with a significant increase in all-cause mortality, but to a lesser extent with disease-free survival. The authors demonstrated by subgroup analysis that the significant association between the pretreatment NLR and the prognostic utility of NLR was more evident among White versus Asian patients [26]. In agreement with that finding, Koh *et al*., [32] analysed in patients of different Asian ethnic groups and reported a significant association between high pretreatment NLR and overall death among BC patients.

## Correlations between NLR and clinicopathological features

A number of studies have suggested an association between the NLR and the prognosis of cancer patients, although no clear explanations have been proposed [34, 35]. Results of previous studies with BC patients with a high NLR presented clinicopathological factors associated with advanced disease, including high histological grade, the presence of a large tumour and a higher T classification [31]. Proctor *et al*. [36], for example, showed in a cohort of 8759 cancer patients (with, at follow-up, 5163 deaths of which 4417 were cancer deaths), that the relationship between thresholds NLR ≥ 4.0 and survival in BC patients (*n* = 621) assessed before and after diagnosis, adjusted for age, sex and deprivation, and stratified by tumour site were associated with shorter overall and cancer-specific survival.

Considering the significance of NLR in correlating with clinical outcomes, Noh *et al*., [22] demonstrated that patients with NLR equal to or higher than 2.5 were associated with increased T stage, younger age, and positive HER2 status. This finding is consistent with the findings in the study with patients with operable breast cancer by Azab *et al*., [37]. The study evaluated the prognostic factor of the NLR in 400 BC patients, showing that patients with a higher NLR were older, had more lymph node involvement and metastases. Dirican *et al*. showed that tumour depth (pT), nodal status, AJCC staging (increasing pathological stage), and distant metastasis status were found to be statistically significant associated with high NLR [16]. On the other hand, Ulas *et al*. [38] did not identify any significant correlations between clinic and pathological parameters or survival and the NLR in patients with BC.

In summary, these results suggest that the inflammatory components are important triggers of cancer growth. According to Proctor, this is consistent with the ‘seed and soil’ nature of cancer growth [36].

## NLR and chemotherapy

The first study to demonstrate that the pre-treatment NLR can be used as a predictor of the response to neoadjuvant chemotherapy (NAC) has been reported for Sato *et al*. [39] the study included 83 patients receiving neoadjuvant chemotherapy (cisplatin and 5-FU) before oesophagectomy for oesophageal cancer. The NLR was calculated before chemotherapy, and the response to chemotherapy was then assessed. Results showed that high pretreatment NLR ( ≥ 2.2) and lymph nodes metastasis were independently correlated with poor response. The response rate was 21% in patients with a high NLR ( ≥ 2.2) compared with 56% in the patients with a low NLR ( < 2.2). The authors also observed that high NLR delays the response to chemotherapy.

Four studies have reported the prognostic value of the NLR in BC patients who received NAC. In these studies, it was again reported that a high NLR was associated with increased risk of relapse and mortality. Pistelli *et al*. [40] reported the association between the NLR, DFS, and OS in 90 patients with triple-negative breast cancer (TNBC). They showed that patients with a NLR higher than three had lower DFS and OS than those with a NLR ≤ 3. Consistent with the results of Pistelli’s study, Bozskurt *et al*., [41] detected in their Cox proportional multivariable analysis showed that a high NLR was independently associated with shorter DFS and OS in patients with stage TNBC who had completed treatment.

According to the published data, the NLR could potentially be used to estimate the likelihood of response to NAC. The use of NLR may facilitate the use of NAC therapy in patients with low NLR to reach a better pCR rate and to improve long-term outcomes. This rationale is consistent with the conclusion by Suppan *et al*., [32] that NLR is an independent prognostic marker for BC. These observations suggest that the lysis of tumour cells by agents may be associated with the release tumour-associated antigens. This could help an immune response against the cancer cells, which could be particularly important in patients that had a sensitisation of the immunity against such tumour antigens before the introduction of chemotherapy [42]. This suggests that the NLR can provide prognostic information to predict the usefulness of adjuvant therapy after surgery or NAC.

Additionally, it is important to highlight that different chemotherapy agents may be associated with different grade of neutropenia (as anthracycline- and taxane-based treatments). Patients with neutropenia after NAC can be treated with granulocyte colony-stimulating factors (G-CSF) to increase the release of leucocytes, which may also modify neutrophil and lymphocyte levels. Indeed, the absence of high levels of neutropenia in patients with a high NLR may suggest insufficient drug dosage and/or limited effect on the tumour-associated immunity. To conclude, these results are in line with the long-term benefits of the previously published treatment effect favouring NAC for OS. Additionally, these confirm the usefulness of this association for patients with BC.

## NLR in histological subtypes

Breast cancer is heterogeneous regarding histology, molecular subtypes, and response to treatment. The definition of triple-negative breast cancer (TNBC) applies to all tumours’ that lack the expression of ER and PR (hormone receptors – HR) and HER2. Luminal A and luminal B subtypes and have been shown to have different gene expression, prognosis, treatment response and profiles [43]. Different clinical behaviours and histopathological characteristics of HR+/HER2+ breast cancers and HR−/HER2+ breast cancers have been reported [44]. In this context, Koh *et al*., [31] in a study of 157 HR+ HER2-BC patients treated with neoadjuvant chemotherapy (mean follow-up of 21 months after surgery), showed that a NLR higher than 2.25 was associated with shorter recurrence-free and overall survivals. A high NLR was an independent risk factor for recurrence and mortality.

TNBC patients usually have a worse outcome compared with those with other BC types owing to a more aggressive pathological behaviour [44]. Recent works have shown that systemic inflammatory parameters could be complementary in predicting TNBC patients’ outcomes. Pistelli *et al*. [40] evaluated the correlation between the NLR before treatment, DFS, and OS in 90 patients with early TNBC. They showed that patients with a NLR higher than three had shorter DFS and OS than those with a NLR ≤ 3. Asano *et al*., [45] related NLR < 3 was associated with prognosis in TNBC patients after successful NAC, older age and post-menopausal status, suggesting that low NLR may help as a biomarker in these patients. Jia *et al*. [46] reported that a high NLR before treatment was independently associated with a worse prognosis for BC and TNBC. One possible explanation for those findings might be the genomic instability in TNBC and a higher tendency to generate neoantigens, suggesting a heterogeneous pattern of immune infiltration [47].

Luminal A tumours are ER and/or PR positive, with a Ki67 level of < 14%. Noh *et al*. [22] examined the NLR and its prognostic implications for specific subtypes. In their study, they showed that an elevated NLR was significantly associated with poorer prognosis for the luminal A subtype. They concluded that each tumour type has its own degree of infiltration of T cells and any prognostic information should integrate the differential gene expression. As a consequence, these differences may be seen as explanations for the various infiltrations of T cells seen in different types of BC, defined by gene expressions [48].

## NLR and NSAIDs use as predictors of survival

Recent studies show that use of non-steroidal anti-inflammatory drugs (NSAID) is possibly associated with a relevant, even if modest, reduction in BC occurrence [49]. Additionally, NSAIDs has been shown as beneficial in the prevention of other cancers [50]. In certain cases, inflammation is clinically clear at the earliest stages of neoplastic progression, accelerating cancer cells genetic evolution and contributes to tumour immune evasion. Supporting the notion that the NSAIDs’ beneficial effect would be greater in patients with higher inflammatory response (involving similar pathways), Forget *et al*. [15] explored the NSAIDs effect in BC patients with a NLR > 4. In those patients, it was observed a twofold reduction in the risk of recurrence when using NSAIDs [51]. These results are in line with the findings reported by Choi *et al*., [52] that reported that the NLR was higher in patients with tumour stage II–III than those in stage I. This would suggest a higher inflammatory status and immune dysregulation in patients with advanced NSCLC.

## Conclusion

The NLR has the potential to be a sensitive prognostic marker. However, prospective studies are still needed to further evaluate its benefits and clinical relevance. In BC, the mechanisms underlying high NLR and the response to anticancer treatment should also be further explored. More clarifying findings on NLR and its correlation with BC prognosis would facilitate the selection of patients who are more likely to benefit from drugs or specific surgical approaches. Evidence is evolving for the NLR prognostic role, but the optimal cut-off levels for NLR remains to be established. A cost-effective analysis and accessibility should be taken into consideration when selecting a laboratory prognostic biomarker, which qualifies the NLR as an excellent one. Currently, the literature suggests that, in patients with BC, the NLR can be potentially used as an independent prognostic marker for DFS and OS in general and should be assessed prior to each type of surgical and drug treatment.

## Figures and Tables

**Table 1.** Main characteristics of all studies included in the review.

First author; publication year; country of origin	Study period	No. patients Low/high NLR	Treatment received	Follow-up	Age (median – ys)	No of distal metastasis	No of deaths	Type of study	Cut-off	Survival analysis	Significant variables
Azab *et al*., 2012; USA	2004–2006	**NLR < 1.8** – *n* = 77 **1.80 ≤ NLR < 2.45** – *n* = 80 **2.45 ≤ NLR < 3.33** – *n* = 78 **NLR ≥ 3.3** – *n* = 81	Surgery	1–7 ys	59, 3/60, 8/60, 8/67	NR	**NLR < 1.8** 1 y – 0 ; 2y – 4% 5 y – 13% **NLR** ≥ 3.3 1 y – 16% 2 y – 25% 5 y – 44%	Retrospective	**Multiple cut-off** NLR < 1.8 1.80 ≤ NLR < 2.45 2.45 ≤ NLR < 3.33 NLR ≥ 3.3	Long-term mortality	Older age Tumour size Nodal status Clinical staging
Forget *et al.,* 2013; Belgium	2010	172 – centre 1 162 – centre 2 - (Ketorolac or diclofenac use – 20-30 mg/75 mg)	Surgery	24 mo	NR	**Centre 1** **NLR < 4 –** 6.3% **NLR ≥ 4** – 16.5% **Centre 2** **NLR ≥ 3** 11.6% **NLR < 3 –** 2, 2%	**Centre 1** **NLR <** **4 –** 2.4% **NLR ≥ ** **4** – 8.2% **Centre 2** **NLR ≥ ** **3 –** 7.9% **NLR < ** **3 –** 3.4%	Retrospective	5 < NLR ≥ 5 4 < NLR ≥ 4	DFS; OS	Age Lymph node invasion
Azab *et al*., 2013; USA	2004–2006	**NLR < 1.8** – *n* = 104 **1.8 ≤ NLR < ** **2.4** – *n* = 106 **2.4 ≤ NLR < ** **3.3** – *n* = 108 **NLR ≥ 3.3** – *n* = 119	Surgery	48 mo	63,6	**NLR < 1.8 –** 2 **NLR ≥ 3.3 –** 24	**NLR <** **1.8 – 1** (1.9%) **NLR ≥ ** **3.3** – 37 (44%)	Retrospective	**Multiple cut-off** NLR < 1.8 1.8 ≤ NLR < 2.4 2.4 ≤ NLR < 3.3 NLR ≥ 3.3	OS	Age Clinical staging (I-III) Radiotherapy
Noh *et al*., 2013; Korea	2000–2010	**NLR < 2.5** (*n* ≤ 327); **NLR ≥ 2.5** (*n* = 115)	NR	5,9 ys	50	**NLR < 2.5 –** 43 **NLR ≥ 2.5 –** 16	**NLR <** **2.5** – 17 **NLR ≥ ** **2.5** – 15	Retrospective	**Multiple cut-off** NLR < 2.5 (*n* = 327); NLR ≥ 2.5 (*n* = 115)	DFS	Nodal status Age ER Luminal A subtype
Forget *et al.*, 2014; Belgium	2003–2008	720	Surgery	69,8 mo	56/60	36	37	Retrospective	3.3 ≤ NLR > 3.3	DFS; OS	Tumour size NSAIDs
Dirican et al., 2014; Turkey	2006–2011	NLR < 1 – n = 60 1.0 ≤ NLR < 2.0 – n = 698 2.0 ≤ NLR < 3.0 – n = 458 3.0 ≤ NLR < 4.0 – n = 170 4.0 ≤ NLR < 5.0 – n = 65	Surgery and multiple-therapy	30 mo	NR	90	–	Retrospective	Multiple cut-off 1.0 ≤ NLR < 2.0 2.0 ≤ NLR< 3.0 3.0 ≤ NLR < 4.0 4.0 ≤ NLR < 5.0	OS; DFS	Tumour depth (pT) Nodal status Clinical staging Metastasis
Cihan *et al*., 2014; Turkey	2005–2010	**NLR <** **3** – *n* = 228 **NLR ≥ ** **3.3** – *n* = 122	Surgery and multiple-therapy	5 ys	55,3	45	–	Retrospective	3.0 ≤ NLR ≥ 3.3	DFS; OS; PFS	Histopathological diagnosis Perinodal invasion Tumour stage HER2 subtype Age
Nakano *et al*., 2014; Japan	2001–2011	**NLR <** **2.5** – *n* = 120 **NLR ≥ ** **2.5 – ***n* = 47	Surgery	85,8 mo	58,9/55,1	**NLR < 2.5** – 15 **NLR ≥ 2.5 –** 14	**NLR < 2.5 –** 9 **NLR ≥ ** **2.5 –** 10	Retrospective	2,5 < NLR ≥ 3.3	DFS; OS	Histological grade Perinodal invasion BMI
Koh *et al.*, 2014; Korea	2002–2010	**NLR ≤ 2.25** – *n* = 91 **NLR > 2.25 – ** *n* = 66	Surgery and multiple-therapy	21 mo	44	25	–	Retrospective	**Multiple cut-off** NLR ≤ 2.25 NLR > 2.25	DFS; OS	Clinical staging (I–III)
Yao *et al*., 2014; China	2009–2011	**NLR ≤ 2.57** – *n* = 496 **NLR > 2.57** – *n* =	Surgery	5,9 ys	50	NR	NR	Retrospective	2.57 ≤ NLR > 2.57	DFS	Nodal status HER2 subtype Diabetes
Pistelli *et al*., 2015; Italy	2006–2012	**NLR ≤** **3** – *n* = 73 **NLR >** **3** – *n* = 17	Surgery and multiple-therapy	53,8 mo	53	**NLR ≤ 3 –** 8 **NLR > 3 –** 5	**NLR ≤ 3** – 4 **NLR > 3** – 4	Retrospective	NLR ≤ 3 NLR > 3	DFS; OS	Age, menopausal status, tumour size, lymph nodes status, Ki-67, necrosis and lymphovascular invasion
Koh et al., 2015; Asia	2000–2008	**NLR ≤** **1.39** – n = 226 **1.39 < NLR ≤** **2** – *n* = 379 **2 < NLR ≤** **2.58** – *n* = 305 **2.58 < NLR ≤** 4 – *n* = 331 **NLR ≥ 4** – *n* = 194	Surgery and multiple-therapy	Patients were followed up in the breast clinic (1 February 2014)	56/53/50/49/50	**NLR ≤** **1.39 – 15** **1.39 < NLR ≤ 2 –** 30 **2 < NLR ≤** **2.58 – 37** **2.58 < NLR ≤ 4** – 54 **NLR ≥ 4** – 61	**NLR ≤** **1.39** – 75 **1.39 <** **NLR ≤ 2 –** 135 **2 < NLR ≤ 2.58 –** 113 **2.58 < NLR** ≤ **4 –** 158 **NLR ≥ 4 –** 118	Retrospective	**Multiple cut-off** NLR ≤ 1.39 1.39 < NLR ≤ 2 2 < NLR ≤ 2.58 2.58 < NLR ≤ 4 NLR ≥ 4	OS	Age; no. of positive lymph nodes; metastasis; HER2 status
Ulas *et al*., 2015; Turkey	2009–2014	**NLR <** **2.38** – *n* = 119 **NLR >** **2.38 –** *n* = 68	Adjuvant transtuzumab	26 mo	51,4	**NLR <** **2.38** –15 **NLR >** **2.38** – 11	–	Retrospective	2.38 < NLR > 2.38	DFS; OS	No significant correlations between NLR and clinico-pathological factors.
Asano *et al*., 2015; Japan	2007–2013	**NLR <** **3** – *n* = 58 **NLR >** **3 –** *n* = 119	Preoperative chemotherapy	3.4 y	56	–	–	Retrospective	NLR < 3 NLR > 3	DFS; OS	Younger age; premenopausal status; pCR result; TNBC phenotype
Hong *et al*., 2015; China	2009–2010	**NLR <** **1.93** – *n* = 298 **NLR >** **1.93** – *n* = 189	Surgery	55 mo	55	48	5	Retrospective	1.93 < NLR > 1.93	DFS; OS	TNBC phenotype
Jia *et al*., 2015; China	2000–2010	**NLR >** **2** – *n* = 804 **NLR ≤** **2 –** *n* = 766	Surgery and multiple-therapy	79 mo	47	242	108	Retrospective	NLR > 2 NLR ≤ 2	DFS; OS	Age, node status; TNBC phenotype
Suppan* et al*., 2015; Austria	2001–2012	**NLR=** 4.97 **NLR =** 2.63 **247**	Surgery and multiple-therapy	123 mo	52	–	–	Retrospective	NLR = 4.97 NLR = 2.63	DFS	TNM (pN1; pN2 + pN3)
Bozkurt *et al*., 2015; Turkey	2002–2013	**NLR ≤ 2 =** 33 **NLR > 2 =** 52	Surgery and multiple-therapy	60 mo	50	–	–	Retrospective	NLR > 2 NLR ≤ 2	DFS; OS	Age, menopausal status, tumour size, lymph node status, grade, Ki-67.

Abbreviations: BMI: body-mass index; DFS: disease-free survival; ER: oestrogen receptor; Mo: months; OS: overall survival; PFS: progression-free survival; NLR: neutrophil-to-lymphocyte ratio; NR: not recorded; NSAIDs: non-steroidal anti-inflammatory drugs; Ys: years; pCR: pathological complete response; TNBC: triple-negative breast cancer.
